# Veterans’ views on alcohol use and experience of alcohol withdrawal syndrome treatment and subsequent alcohol use disorder care at two highly rural-serving VA hospitals: a qualitative study

**DOI:** 10.1007/s10865-026-00687-x

**Published:** 2026-07-09

**Authors:** Tony Pomales, Daniel Ball, Jeydith Gutierrez, James C. Willey

**Affiliations:** 1 Veterans Rural Health Resource Center-Iowa City (VRHRC-IC), Iowa City Veterans Affairs Health Care System, VA Office of Rural Health, Iowa City, IA USA; 2 Center for Access and Delivery Research and Evaluation (CADRE), Iowa City Veterans Affairs Health Care System, Iowa City, IA USA; 3 Division of Hospital Medicine, Department of Internal Medicine, University of Iowa Carver College of Medicine, Iowa City, IA USA; 4 Department of Internal Medicine, University of Utah, Salt Lake City, UT USA

**Keywords:** Veterans, Alcohol withdrawal syndrome, Alcohol use disorder care, Barriers and facilitators, Qualitative

## Abstract

Alcohol use disorder (AUD) is a significant cause of morbidity that disproportionately affects veterans and rural-serving hospitals. As part of a larger quality improvement project to improve care for veterans with AUD admitted to rural-serving Veterans Affairs (VA) hospitals, we conducted a qualitative study using a phenomenological research approach to incorporate the veteran perspective. Participants were *n* = 16 veterans with AUD and recent admissions to two highly rural-serving hospitals. Veteran views and experience were elicited by conducting 30–60 min sessions of semi-structured interviews and assessed using thematic content analysis. Two themes relating to participants’ views on alcohol use were identified: perceived unmet need, resulting in the use of alcohol to treat pain and cope with trauma and co-occurring mental health symptoms, often related to military service, and factors influencing hospital admission and need for additional treatment services. Themes relating to participants’ experience of AWS treatment and subsequent AUD care were identified and divided into barriers and facilitators. Barriers included: limited, uneven distribution of hospitals; perceived premature discharge from inpatient AWS treatment; perceived discriminatory treatment and stigma about AUD; and a shortage of VA residential rehabilitation treatment programs. Veterans’ geographic location and the uneven distribution of RRTPs within large catchment areas restricted access and increased travel burden. Preference and excess demand for VA RRTPs and limited service capacity contributed to long wait times. Facilitators included clinician support and spousal/family and veteran/peer support. Our findings suggest that a non-judgmental treatment environment and improved access to specialized services are essential to receiving effective AUD treatment, particularly for veterans in rural areas who have been repeatedly admitted for AWS. The VA should examine options to reduce stigma, align treatment with patient goals, leverage peer-led programs, and promote comprehensive care that addresses co-occurring conditions and underlying causes of alcohol misuse.

## Background

Alcohol use disorder (AUD) is a chronic, relapsing medical condition in which a person has difficulty stopping or controlling alcohol use despite negative effects on their social life, work, or health (National Institute on Alcohol Abuse and Alcoholism [NIAAA], 2025). Excessive alcohol consumption, binge drinking (greater than 4 drinks for women and 5 drinks for men in one occasion), and heavy drinking (8 drinks or more for women and 15 rinks or more for men in one week) is associated with over 175,000 Americans deaths annually in the US (Esser et at, 2024). Veterans and those living in rural areas have a higher prevalence of AUD (Wagner et al., 2007; SAMHSA, 2020) and face more challenges accessing treatment in their local communities. Several studies have described multiple factors, including limited addiction medicine specialists, geographic barriers, lack of specialized treatment centers, and negative perceptions of medication-assisted treatment (Browne et al., 2016; Davis and O’Neill, 2022; Edmonds et al., 2021; Najjemba, 2025; Pullen & Oser, 2014; Richard et al., 2020).

Alcohol withdrawal syndrome (AWS) is characterized by autonomic hyperactivity and central nervous system activation that develops following a reduction or cessation in alcohol intake (5th ed., DSM-5, American Psychiatric Association, 2013; Stehman & Mycyk, 2013). AWS is frequently treated in the hospital due to the risk of life-threatening complications such as seizures and delirium tremens (DTs) (Hall & Zador, 1997). Veteran hospitalizations for AWS are a key opportunity to diagnose and treat underlying AUD, but inpatient providers often focus on treating acute AWS symptoms and deferring AUD management for outpatient care. This short-term approach to the inpatient management of AUD is evident in the low prescribing rates of AUD medications on discharge after AWS treatment, ranging from 7 to 26% in community settings (Bernstein et al., 2023; Ober et al., 2025), to 50.7% in veterans (Allaudeen et al., 2024). There is also a low rate of linkage to SUD treatment after discharge, which occurs in < 50% of patients admitted with AWS, both among veterans and non-veteran populations (Allaudeen et al., 2024; Ober et al., 2025). The inability to appropriately initiate therapy for AUD in the inpatient setting is a missed opportunity to address the serious consequences and poor outcomes associated with AUD. Our previous work has shown veterans admitted to U.S. Department of Veterans Affairs (VA) hospitals with any AUD-related diagnoses had a high 5-year mortality (~ 35%) (Willey et al., 2025). Despite this high mortality, only about 15% of patients with AUD diagnosis receive treatment (Mekonen et al., 2021).

Incorporating veteran perspectives on alcohol use and hospital care for AWS and AUD is crucial to identify and address gaps in AUD treatment, particularly immediately post-discharge, which is a vulnerable and stressful time in which patients have a high risk of relapse (Becker, 2008). This elevated risk of relapse is evidenced by high readmission rates (25%) and 43% return to heavy drinking for veterans admitted with AWS at 30-day, while abstinence rate was only 17.8% at 6 months (Allaudeen et al., 2024). One study found the highest hazard was concentrated in the first 3 months post-discharge (Malarmathi et al., 2025). Previous qualitative research on patients using alcohol and other substances in community settings has exposed several actionable barriers to care that are often invisible to traditional treatment metrics. For example, long wait times between assessment and treatment initiation create critical gaps in care, in which patients are often waitlisted without ongoing support (Francia et al., 2023). Also, patients recognize a greater need for treatment during symptomatic periods, while providers wait for most symptoms to subside before initiating AUD treatment. (Kim et al., 2025; Tomlinson et al., 2025). This creates a mismatch in patient and provider priorities. Finally, limited awareness and misconceptions regarding AUD medications both from patients and providers (Kim et al., 2025; Tomlinson et al., 2025) may contribute to low AUD treatment rates.

As part of a comprehensive multi-year and multi-site quality improvement (QI) project to improve care for veterans with AUD admitted to rural-serving VA hospitals, we conducted a qualitative study to enhance our understanding of the VA patient experience of AWS and AUD treatment and assess barriers, facilitators and veteran-specific factors influencing access to and continuation of therapy for AUD. To our knowledge, there are no other qualitative studies in the veteran population assessing these issues. Cheney et al. (2018) conducted a mixed methods study among veterans to identify barriers to accessing mental health care, including alcohol use. While the study was helpful in identifying barriers to utilizing VA mental health services from the perspective of VA patients (e.g., a stigmatizing ethos towards mental health and mental health service use in the military; distrust of government institutions; transportation barriers), it did not explore veteran views and experiences of hospital care for AWS, nor did it address specific barriers and facilitators to accessing AWS treatment and continuation of therapy for AUD. This specific focus is critical because inpatient and immediate post-discharge periods represent unique windows for intervention during a vulnerable time, with high relapse risk. Our study objective was to better understand the veteran experience of AWS treatment and subsequent AUD care, and identify barriers and facilitators, at the individual and at the health system level, that could inform tailored, patient-centered interventions to enhance access to and quality of AWS treatment and AUD care among veterans served at rural VA hospitals.

## Methods

### Research aims and study design

This qualitative study was conducted as part of a VA Office of Rural Health-funded QI project aimed at: (1) examining regional variations in AUD-related hospitalizations, readmissions, and outcomes among rural-serving VA hospitals, and (2) identifying opportunities to improve care and disseminate best practices for the management of AWS and AUD across rural-serving VA facilities. We used a phenomenological research approach (Rodriguez & Smith, 2018; Shorey & Ng, 2022) to understand the lived experience of veterans with AUD and recent admissions to two highly rural-serving VA hospitals, with the goal of identifying areas to improve care. The data were collected via semi-structured interview and analyzed using thematic content analysis. We chose to be guided by a phenomenological research approach, as it is an appropriate way to understand the patient experience, barriers and facilitators to care, and how people feel about, live with, and/or manage health-related phenomena (see Davoodi et al., 2022; Finlay, 2003; Rodriguez & King, 2009).

### Participants and sampling

Eligible participants were identified from a database of patients who had an inpatient admission from January 2022 through April 2024 with an ICD-10 diagnosis code of AWS at two rural VA participating sites. Based on these criteria, we had a sample of 31 and 32 potential participants for sites 1 and 2, respectively. Sampling sites were chosen based on their involvement in our ORH funded quality improvement project to improve AWS care in rural VAs. Both sites were in the Midwest and considered rural (i.e., more than 50% of the patients served live in rural or highly rural areas). Patient and site rurality was defined using rural-urban commuting area codes, developed by the Department of Agriculture and the Department of Health and Human Services (USDA ERS, 2024). Due to the small number of patients meeting our eligibility requirements, we do not describe the sites further to ensure privacy.

Using purposive sampling, all eligible patients were invited via telephone or email to participate in the study. Of the *n* = 64 eligible patients, 24 did not answer our call or respond to our email after three attempts to be reached. Seven had a wrong/disconnected number or a calling restriction. Five people did not answer or call back after we had scheduled an interview with them. Eleven declined to participate and cited different reasons for doing so, including lack of time/interest and an inability to participate due to medical reasons or not wanting to recall their experience. A total of sixteen (*n* = 16) eligible patients were enrolled in the study. No one on the research team had any pre-existing relationships with any eligible patients or enrolled participants.

### Interview and data collection

Participants were told the purpose of the interview was to better understand veterans’ experiences of treatment for alcohol-related problems, with the goal of improving management of AWS and AUD in rural VA hospitals. Seeking to describe veteran mental models of AWS treatment and subsequent AUD care, we specified that we were particularly interested in how they themselves viewed these issues, how they managed AWS and AUD, barriers faced, and how they felt they were treated when seeking care for AWS and AUD.

Two project researchers with content experience and expertise in ethnographic methods and qualitative research and analysis (T.P. and D.B.) conducted and recorded interviews using Microsoft Teams. T.P. and D.B. developed the interview guide, with input and guidance from J.G. and J.W. as specialized medical experts. Interviews began with more general questions about participant’s health, alcohol use, drinking motives, and VA services used. As participants became more comfortable and spoke more freely, we asked about experiences with AWS treatment (both inpatient and outpatient) and AUD care (e.g., rehabilitation services, medication treatment), any access challenges faced, and followed up delicately whenever they spoke of stigma. Each interview lasted approximately 30–60 min in order to collect the required data. Participants were compensated $50 for their time.

### Data analysis

Auto-generated interview transcripts were cleaned for readability and reviewed for accuracy. Thematic content analysis as described by Braun and Clarke (2006) was performed to identify major themes regarding veterans’ views of alcohol use and experience of AWS treatment and subsequent AUD care. This involved gaining familiarity with the data by reading and re-reading transcripts. After identifying significant statements, T.P. and D.B. decided on combining a deductive and inductive approach to coding the data (Fereday & Muir-Cochrane, 2006). We used Cooper’s (1994) model for drinking motives to sort participants’ views on alcohol use into initial categories. An inductive approach was used to identify preliminary codes that could be categorized and sorted into themes. T.P. and D.B. started data coding by independently reviewing three transcripts and identifying preliminary codes based on significant statements. An initial codebook was developed in consultation with the entire research team (T.P., D.B., J.G., J.W). Next, T.P. and D.B. independently applied the preliminary codebook to two transcripts to assess the codebook’s utility and establish intercoder reliability. After finalizing the codebook, T.P. and D.B. imported the transcripts to MAXQDA (1989–2025, VERBI Software. Consult. Sozialforschung GmbH, Berlin, Germany) and carried out systematic coding. Codes were then organized into potential themes, which were reviewed, refined, and named through iterative discussions with the entire research team. We did not request feedback from veteran participants at any stage of the analysis process. However, J.G. and T.P. did consult a rural health-focused veteran engagement panel on how best to disseminate study findings to veterans.

### Ethical issues

This study was reviewed and approved by the University of Iowa Human Subjects Office/Institutional Review Board (IRB) and the Iowa City VA Healthcare System Research and Development Committee and determined to be non-human subject research as part of a quality improvement effort. The consolidated criteria for reporting qualitative research (COREQ) guidelines have been followed in the reporting of this data (Tong et al., 2007). The authors had full access to and take full responsibility for the integrity of the data. In order to keep participants anonymous and to distinguish them from one another in the presentation of our findings, a study ID was assigned to each participant. Informed consent was obtained prior to each interview.

## Results

Sixteen (*n* = 16) veterans completed interviews. The average age of participants was 50 years (range 33 to 78) and predominantly male (*n* = 13), White (100%), retired/unemployed/disabled (*n* = 13), and had some college or greater education (*n* = 13); see Table 1 for more detailed demographics.

**Table 1:** Demographic characteristics of study participants

Characteristic	*n or M* (*n* = 16)^a^	% or Range
Age in years, *M* (range)	50	33–78
*Sex*		
Men	13	87
Women	2	13
*Race*		
White	15	100
*Education*		
High school diploma	3	20
Some college/associate’s degree	7	47
Bachelor’s degree	2	13
Master’s degree	3	20
*Marital status*		
Single/divorced/separated	9	60
Married	6	40
*Sexual orientation*		
Heterosexual	15	100
*Military branch* ^b^		
Army	7	54
Marines	2	15
Air force	2	15
Navy	2	15
*Employment status*		
Employed	3	20
Unemployed	6	40
Disabled	3	20
Retired/semi-retired	3	20

^a^We were unable to collect demographic information for one participant due to time constraints during the interview

^b^We did not collect military branch information from two participants

A total of eight unique were extracted from the data. Two themes representing participants’ views on alcohol use were identified, including: (1) perceived unmet need, resulting in the use of alcohol to treat pain and cope with trauma and co-occurring mental health symptoms, and (2) factors influencing hospital admission and need for additional treatment services. Six themes emerged regarding participants’ experience of AWS treatment and subsequent AUD care and divided into barriers and facilitators. Barriers included: (3) limited, uneven distribution of hospitals, (4) perceived premature discharge from inpatient AWS treatment, (5) perceived discriminatory treatment and stigma about AUD, and (6) a shortage of VA residential rehabilitation treatment programs within large catchment areas. Facilitators to AUD treatment included: (7) clinician support and (8) veteran/peer and spousal/family support.

### Theme 1: perceived unmet need: using alcohol to treat pain and cope with trauma and co-occurring mental health symptoms

Two themes representing participants’ views on alcohol use were identified. Theme 1 identified a common thread of perceived unmet need in pain management and mental health care. Participants with chronic pain frequently mentioned feeling like they had to manage their pain on their own, as did those who cited mental health concerns like PTSD and depression. Participants disclosed using alcohol to treat pain and cope with trauma and co-occurring mental health symptoms.

“I have a couple drinks just to help me get through [the pain]. It’s the only thing I found that helps. Medicine don’t help for s**t. Painkillers don’t do anything for me…I was trying to quit or slow down and with PTSD…” (10025).

Participants often identified military service and combat as the source of these issues, as indicated in the quote below:

“…something is triggering my PTSD side. I need to numb that out, so there’s not a pill for that, not that I know of yet…my primary objective is to live with the symptoms that I experience from my mental health diagnosis of PTSD and major depression…the traumas I have experienced, they revisit me like ghosts. And when they do pop up, I don’t wanna turn to alcohol for the treatment. I wanna be able to cope with it [instead of] treating my problem with another problem.” (10023).

Participants who cited treating pain as their primary motive for drinking described how they gradually lost the ability to stop or control their alcohol intake and got stuck in an unhealthy cycle of heavy drinking. One participant stated that his drinking “started out managing pain” after several “non-surgical procedures” and a desire to quit his pain medicine, but over time his drinking intensified and became compulsive:

“…when I finally had had enough of the pain medications…I didn’t quite have as much pain, and drinking, I didn’t see it coming, how…eventually it was every day. By then, it’s too late. You realize it’s a disease.” (10006).

Another common view shared by participants was the use of alcohol to cope with the co-occurrence of pain and mental health symptoms. Participant 10,026 described how the broader impacts of the COVID-19 pandemic worsened his mental health and combined with the physical pain “present in [his] life every day” to become “a perfect storm” that led him into “a downward depression and subsequent drinking and abuse of alcohol.”

Participants who identified using alcohol to cope with comorbid pain and mental health symptoms frequently cited PTSD as a mediator and source, highlighting the veteran-specific nature of their emotional distress. Participants identified co-occurring pain and PTSD as a major risk factor that could lead to heavier and heavier drinking if untreated. Like Participant 10,026, Participant 10,019 described how his alcohol use started by trying to treat his pain, but eventually became a strategy for coping with the PTSD that developed after surviving a traumatic event in combat:

“[It] started with pain, but it was also mentally involved. … I had a lot of PTSD and survivors’ guilt from an IED explosion. And I can’t go to sleep…I’d do a shot [of alcohol] at night just trying to help me sleep, and after a while, a shot wasn’t enough…eventually, it was getting to a bottle a night.” (10019).

Although participants were aware of the negative consequences of self-medication with alcohol and frequently mentioned wanting/trying to stop or reduce their drinking, they often felt like effective treatment was beyond their reach. This was an especially salient point among participants with co-occurring pain and PTSD.

### Theme 2: factors influencing hospital admission and need for additional treatment services

Theme 2 identified severe presentations of AWS as the main factor influencing hospital admission and need for additional treatment services. Chronic heavy drinking, as described above, increased participants’ risk of AWS. Many participants described life-threatening complications when they tried to substantially reduce or abruptly stop their intake:

“…since I’ve been using alcohol for a while, I get the DTs, the Delirium Tremens…the hallucinations…the vomiting, extreme dehydration, and then hands and arms start cramping up.” (10019).

“I get super shaky. I get really sweaty. Like really nauseous. I’ve had a history of seizures. …I have tactile hallucinations.” (10028-2).

Many participants disclosed repeated AWS episodes, repeated visits to the Emergency Department, and repeated AWS admissions. Participants who repeatedly retuned to alcohol use after AWS treatment were especially aware of the need for inpatient detoxification when they tried to substantially reduce or abruptly stop their intake. These participants did not view home detoxification as a safe option. As one participant said,

“I’ve had seizures, Tremens, sweats, insomnia, hyperventilating paranoia…everything but jaundice…My chronic relapse cycle would end in that level of alcohol usage that would require hospitalization in order to safely come off of alcohol. Otherwise, if I were to try to detox at home, I would be a good candidate for a funeral.” (10023).

Notably, we found that participants residing in more remote rural areas occasionally took preemptive steps to avoid an unsafe withdrawal at home, by presenting themselves at the hospital for inpatient treatment before AWS symptoms set in. As one participant stated,

“I usually know from experience that I’ve been drinking too much too long and that it’s time to detox…The symptoms are pretty much cognitive more than physical…I know the danger of cold turkey…And since I live alone and out in the rural community, I don’t want to be in that situation.” (10018).

Participants identified other factors influencing hospital admission and need for additional treatment services, including worsening of cognitive and motor functioning and other alcohol-related health complications such as alcohol-induced hepatitis. When the situation became intolerable or life-threatening, they often sought addiction treatment or intensive care services to prevent more severe health consequences, including death. As two participants stated,

“Well, what made me come [to substance use treatment] was I was at the point where I was having to have a drink first thing in the morning just to stop the shakes…I got tired of it.” (10024).

“I had to go into the VA hospital…I spent about 2½ months there. I had acute alcohol, um, I think they call it hepatitis…but it almost killed me…I’m just slowing recovering…it was pretty much all alcohol related.” (10020).

Adverse occupational and legal consequences and strained family dynamics were also identified as factors influencing need for additional treatment services.

“I had a DUI, so I actually had to go see a therapist.” (10005).

“[F]or me, life became unmanageable, and I was about to lose it all: the house, the family, my job, my sanity.” (10023).

### Theme 3: barrier: limited, uneven distribution of hospitals

Six themes representing barriers to AWS treatment and subsequent AUD care were identified. Theme 3 identified that a major barrier to inpatient AWS treatment for participants living in rural areas was the limited and uneven distribution of hospitals. Participant 10,019, for example, noted that “the nearest hospital to me is [VA hospital name], which is probably an hour and 20 minutes away.” These participants identified high costs and significant travel burden getting to and from a hospital as negatively impacting their ability to detox inpatient, under medical supervision. For some, the high cost of taking a taxi to and from a hospital sometimes led them to take greater risks with their health. As one participant explained,

“…because of my experience living in [area name], the rural part of it is a challenge, getting to and from detox is a bit of an issue. … I have driven up there [i.e., VA hospital location] at times, and I shouldn’t have been driving. But it’s…54–55 miles and its $100 to get back. (10018)

Those who experienced acute AWS and other life-threatening disease processes faced unique geographical challenges. The complex care required for severe presentations of AWS coupled with other life-threatening disease processes meant participants who had already traveled a significant distance to the nearest hospital would frequently need to be transferred to another hospital, sometimes in another state via ambulance, to receive specialized treatment or a higher level of care. As one participant observed,

“…treatment [in highly rural area location] is hard because there’s no treatment for us…they have nowhere to detox you…There’s no set path forward for detox…you’re gonna get into an ambulance and they’re gonna bring it to [city name in a different state] or [city name in a different state]. So, I went to the [VA hospital location] and they actually sent me to a civilian hospital…my level of alcoholism was really bad…they didn’t have life support facilities.” (10006).

### Theme 4: barrier: perceived premature discharge from inpatient AWS treatment

Theme 4 identified a mismatch between patient and provider priorities regarding discharge from inpatient AWS treatment that led to patient dissatisfaction and, in some cases, negative patient outcomes. Several participants recalled situations where they felt prematurely discharged from inpatient AWS treatment, that is, before they felt like their AWS symptoms were fully treated or managed:

“When they discharged me, I went home, and I was still starting to vomit, and I was still not able to keep any food down, and, well, that note they gave me is just if it persists, come back to the hospital, and I ended up having to do that because I was still so dehydrated…it felt like they were trying to get me out of there.” (10019).

Although hospitals are only paid to treat the acute emergency of AWS inpatient, several participants identified feeling rushed to discharge without a clear plan or path forward for continued AUD care:

“They pretty much just wanted to shuffle me out the door…it wasn’t very personalized…they really didn’t refer me to any aftercare…It was really just to get me stabilized and out the door…They really didn’t give me any follow-up.” (10020).

Participant 10,027 recalled two cases of perceived early, clinician-directed discharge that he opposed, and which influenced a return to drinking on the second. In the first case, he called an ambulance to take him to the nearest hospital for inpatient AWS treatment, which happened to be a non-VA, community hospital. He left feeling unsatisfied and feeling like his symptoms were not fully resolved. During the interview, he swore never to return to that hospital, due to a perception that he was discharged prematurely:

“…they just pumped me with some IV and gave me some drugs and medication…around there for like…8 or 10 hours…and then they just released me and sent me home. … it helped some, but it didn’t really do that much, so I’ll never go to that hospital again” (10027).

In the second case he recalled, he was taken to his local VA hospital. Again, he disagreed with the doctor about how long he should stay inpatient. He said he tried to express his health concerns to the doctor but to no avail. The doctor felt that he was ready to be discharged. This did not result in a positive outcome. He returned to drinking immediately after being discharged to, as he said, “feel better”:

“I told the doctor, ‘I’m not ready.’ And he insisted, and I was very disappointed with him. … I tried to just be open and honest, and he just didn’t wanna listen to me. And he just wanted me to get the hell out of there. … I had explained to him. ‘Look,’ I said, ‘It’s gonna take me at least a week for me to feel better, maybe the alcohol’s out of my system, but I’m still not ready.’ And he said, ‘No, the alcohol’s out of your system, you can go now.’ … I didn’t even have my balance…my head was still all cloudy. I couldn’t hardly even walk to the van. And what did I do when I got home? I went and drank again, just so I could feel better.” (10027).

These findings suggest that hospital incentives to reduce length of stay might hinder treatment seeking and full recovery and even cause patients to be readmitted, making the exiting difficult situation of limited hospital options for veterans living in rural areas worse.

### Theme 5: barrier: perceived discriminatory treatment and stigma about AUD

Theme 5 identified perceived discriminatory treatment stigma about AUD as a pervasive barrier among participants that reduced access to care. Participants reported external stigma (i.e., negative attitudes or discriminatory treatment directed toward individuals by others) (Gray, 2002) related to AUD in Emergency Department and inpatient settings, in both VA and non-VA hospitals.

Many described being treated by clinicians and other hospital staff in a moralizing and hectoring manner that caused them to internalize negative messages about themselves, leading to feelings of worthlessness, low self-esteem, and, significantly, a reduced willingness to seek treatment.

“…when you show up in the VA and anything that’s alcohol- or drug-related, they treat you indifferent, coldly, like, get your act together, what’s wrong with you?” (10026).

Participants identified an awareness of alcohol use disorder as a chronic, relapsing brain disease, but noted how this did not necessarily translate into being treated the same as other patients with a chronic disease. As one participant, who used alcohol to cope with the recent losses of her brother to suicide and her mother to COVID-19, observed,

“There’s a Dr. (name) in there who has to lecture me every time I’m there about how I’m slowly killing myself…makes me feel terrible about being there…the last time I was able to stay sober the longest was seven months, and that was because I did not want to go back to (hospital name) ever. …they didn’t treat me properly, and they could also not treat me like I’m a criminal when I go there, because…if the alcoholism is a disease, then they should treat me like it’s a disease, and not give me lectures every time on how I’m not doing things correctly…Dr. (name), told me I was the worst female alcoholic he’d ever had. … it erodes my self-esteem.” (10021).

Participants who reported external stigma in VA hospitals mainly spoke of feeling mistreated by clinicians while seeking inpatient AWS treatment. Perceived discriminatory treatment reported to us included messages directed at participants that reproduced the erroneous assumption that people with AUD have control over their condition and are to blame for it. It also included experiences where clinicians not only failed to show empathy or recognition of AUD as chronic, relapsing brain disease, but also seemed to neglect their responsibility to provide medical care. As the quotes below show:

“I went to go to the VA for help, and I was really intoxicated. I needed to detox, but most importantly, I wanted to find out how they could help me. And the doctor in the yard just—he looked, he talked to me for about five minutes. He gave me an AA pamphlet and said that ‘you’re an alcoholic. There’s no reason the military should be responsible for your care.’ Like, he told me that straight up and, you know, all my friends had just died and stuff…I thought, ‘Okay, there’s nothing.’” (10028-1).

“I had one nurse tell me that he was only going to come into my room one time at the beginning of shift and he didn’t want to see me for the rest of shift, really…He said last time he was in my room a whole bunch of times and he’s not gonna do that again. I don’t even remember the last time he was my nurse, ‘cause I was, like, having withdrawals, you know? … I was like, ‘Oh man, I feel like I need a new nurse. What’s going on here?’ And then he was a shit the whole night through.” (10028-2).

Participants who reported external stigma in non-VA, community hospitals similarly described situations where clinicians and hospital staff used perceived unfavorable language that led to feelings of worthlessness. One participant said he felt like a “second-class citizen” after being labeled a “drunk” by hospital staff:

“I’ve overheard…the staff in the hall in the community [i.e., non-VA] hospitals saying, ‘Oh, we got another drunk in here,’ like super rude about it. … I felt like a piece of shit, felt like I was being treated second-class, because it’s alcohol and it’s not anything like giving birth or something.” (10019).

Although comparison of VA and non-VA hospital settings was not a goal of our study, our analysis did reveal some variations in participant experience regarding perceived discriminatory treatment and stigma in both settings. Some observed that VA hospitals offered a less stigmatizing environment than non-VA hospitals:

“…the staff [at VA hospital name]…they were very compassionate, and they didn’t treat me like a…less-than-person just because of the issues. …it was so much better than the [civilian] hospital…[There, ] I felt like I was…in a mental health institution…But at the VA, I was very well taken care of…And I wasn’t confined to my room.” (10005).

“…before I got involved with the VA, it was terrible. I don’t know if it’s the stigma with like civilian care…but it doesn’t seem to be taken very seriously on the civilian side, to where I do feel like the VA recognizes it as a legitimate issue that needs to be addressed.” (10017).

All in all, these findings suggest that discriminatory treatment and external stigma related to AUD represent a substantial challenge to accessing treatment, particularly at a vulnerable time that may also represent a unique opportunity for intervention.

### Theme 6: barrier: a shortage of VA residential rehabilitation treatment programs

Theme 6 identified a shortage of VA residential rehabilitation treatment programs (RRTP) as a major barrier to AUD treatment, particularly for veterans living in rural areas with repeated admissions for AWS. Participants identified a need for additional treatment services – especially in the critical period following inpatient AWS treatment – but also identified that accessing these services could be particularly challenging in their specific geographic regions. Participants cited different, interconnecting factors that explained the substantial challenge they faced. These included: (1) a limited, fixed number of rehab facilities in the same market as their parent VA medical centers, (2) a large VA catchment area that overlapped with, or spanned across, state lines, meaning that, in a lot of cases, veterans are traveling long distances from their homes to access a VA RRTP, (3) inclement weather, which increased travel burden, (4) feeling isolated from family and the emotional toll of leaving family behind, especially children, for weeks or months at a time, (5) long wait times, and (6) an excess demand for VA RRTPs, which contributed to longer wait times for residential rehab.

“[The RRTP] was just so far away that the thought of it to me was just ridiculous…Six hours in the winter is pretty far.” (10018).

“I’ve always hated that I had to go so far away from home, and I have to leave my kids…I tried to get treatment a couple times at [RRTP name], which is in the city that I live in, and they wouldn’t take me because I’m a veteran, because they don’t take veteran health care.” (10021).

“…up at [RRTP location], only thing I would say is just more opportunity to be closer to your home and have family more connected in the process. Because it would be a 5-hour, 6-hour drive for my family to come up…and I think as you get a clear head, you start to yearn for–I started to miss them…So, I wish there was just a little more availability for inpatient care through the VA…or at least you guys could partner maybe with another local,--give us a little more options…closer to our homes.” (10026).

Participants seeking RRTP services often mentioned two to three potential programs they could attend. However, given the high demand for RRTP services and the limited number of RRTPs and beds available in veterans’ catchment areas, same-day or next-day access was not available. The reported waiting period ranged from 1 to 3 weeks. A common experience for participants was being discharged from inpatient AWS treatment, going back home, and then waiting at least one week before an RRTP opening became available:

“[My mother and grandmother] brought me to [VA hospital name]…I detoxed there. They stabilized me and then they sent me down to…that center in [state name, city name]…for, I think, a week…but I couldn’t get into a substance abuse treatment program. There was a waiting list…so they released me out there. I went and stayed about a week at my parents’ house, and I got a call to go to [RRTP location] about 4 1/2 hours. … I wish we had some more resources.” (10012).

The long wait time frustrated patients and providers, who were especially keen to get their patients into treatment during the critical period following inpatient AWS treatment, when patients may be more motivated to enter treatment but still very vulnerable to retuning to alcohol use. To illustrate using one case, participant 10,021’s providers desperately wanted her enrolled in a RRTP as soon as possible, fearing she might die after repeated visits to the ER in the span of one week. But regrettably, there were no beds available at the RRTP where they sought to transition her care. Despite her grave situation, she was given an admission date a few weeks off. As she said,

“I’ve been struggling lately and going back and forth to [non-VA, community hospital name] like three times in one week and withdrawing kind of hard and then drinking hard. And my counselors [at VA] were like, ‘We want you in treatment like as soon as possible.’ And they’re like, ‘Can you get in and even sooner?’ And I’m like, ‘Well, I called up [VA RRTP]…And they said they could get me in on a certain date…it was like a few weeks away.” (10021).

During interviews, participants voiced a strong preference for VA RRTPs. However, the excess demand for VA RRTPs often extended wait times for veterans in need. Programs with civilians and a large number of enrollees were typically less preferred, but veterans were sometimes left with no other options. One participant who enrolled in a non-VA RRTP was so dissatisfied with the program and the living conditions there that he decided to cut his stay short; and regrettably returned to drinking soon after leaving.

“I ended up in the hospital, and the VA representative came and they put me through this outside [non-VA] treatment in [city name]…They wanted me there for 9 months or something, but the place was horrible, and I ended up leaving, and I ended up…doing the same thing. Then, I end up back in the ER.” (10026).

This participant told us that he ended up at that particular non-VA RRTP because there were no beds available at the smaller, VA RRTP that would have been his providers’ first choice for him. When he was readmitted for inpatient AWS treatment after he returned to drinking, he said,

“VA contacted me, and then they started to -- cause the first time they couldn’t get me in at [VA SUD treatment program location]. There wasn’t any beds open…the second time they made me priority, and I got in.” (10026).

Several participants mentioned repeat stays in residential rehab. Those who were enrolled in different RRTPs (different locations, often across state lines) commonly expressed dissatisfaction at not being able to attend the same RRTP, believing that it would lead to disjointed care. As one participant (10021) said, “the discontinuity of care is ridiculous as far as like going from one place to another.”

### Theme 7: facilitator: clinician support

Two themes representing facilitators to AWS treatment and subsequent AUD care were identified. Although high levels of stigma were reported by participants while seeking treatment for AWS, participants nonetheless identified clinician support as a key facilitator to treatment.

When participants reported positive inpatient AWS treatment experiences they often referred to or even named specific clinicians that interacted with them in a supportive manner and monitored them closely during treatment:

“I couldn’t do a lot [while hospitalized], but I just felt it was like around the clock type care … the doctor… stopped in every day…they were real understanding.” (10020).

Clinicians spotlighted by participants as facilitators were also identified as being skilled at providing regular follow up. To illustrate using one case, during his interview, participant 10028-1 identified several issues – ADHD, combat-related PTSD, marriage difficulties, familial obligations – that influenced his chronic heavy drinking and repeated visits to the hospital for AWS treatment. During one visit to his closest VA hospital (which was about a two-hour drive from his home), he was admitted for three days and, opportunely, linked up with a doctor who “really stepped up” and paved a path to recovery. The doctor scheduled an in-person follow-up appointment after his “three-day detox” and continued to follow up with him electronically via video visits, enabling access to addiction treatment. When he moved to a different state and faced a lapse in care, the doctor “went out of his way to make sure that [he] had support” and access to a new treatment program.

Participants who reported positive experiences in RRTPs spotlighted clinicians who were skilled at listening, showing empathy, and developing patient’s sense of agency to stay the course:

“I don’t think the counselors that I worked with [in rehab] would allow me to give up on myself, even though I offered a lot of push back. …they were trained in the magic arts of being able to give myself a sense of agency and being able to fix myself.” (10023).

“The staff and level of medical care–and they listen …nobody puts you down for [having misused alcohol], that’s for damn sure. Everybody’s there to support you.” (10026).

Participants who were further along in their recovery often spotlighted multiple clinicians, showcasing the benefits of a multidisciplinary, comprehensive approach to treating AUD:

“[M]y new psychiatrist who I just got…she’s really good…I’m back with [social worker name]…she’s really good too, and I’ll be using for social work. And I’ll see my psychiatrist…and one other doctor that’s a prescribing pharmacist. They seem to know the most about the cravings, medications.” (10021).

“I go [to the VA] for treatment for PTSD, major depression, and substance use, and my drug of choice is alcohol. So, I see a counselor regularly…she’s a dual diagnosis counselor for PTSD and substance abuse. My psychiatrist is through the VA, who prescribes my mental health medication, and I attend several support groups that are made by veterans and a peer support or a VA facilitator. I also use the auxiliary programs like Whole Health to have like an accountability coach to keep me goal-oriented, which…does a great deal for keeping me moving forward with mental health goals and not backtracking.” (10023).

### Theme 8: facilitator: veteran/peer and spousal/family support

Theme 8 identified two social supports as additional facilitators to AUD treatment: veterans/peers and spouses/family. For many, connecting to or fostering a relationship with a fellow veteran/peer was important and meaningful. This was especially true for participants with strained family relations. Participant 10020 said he felt “pretty much alienated from [his] family” (i.e., his ex-wife, children, and siblings), and discussed how his “peer-to-peer” became his strongest support and best “resource,” other than his VA social worker:

…my peer-to-peer is real good…I like him because…he didn’t really come down on me or pressure me…‘cause I’ll be honest; I’m not really a big AA person. That hasn’t done much for me in the past.

Participants who attended a VA RRTP often observed that they had more success and better experiences in groups without civilians, because they felt a stronger sense of understanding and shared experience in veteran-only groups. As one participant commented,

“…a lot of [the activities in rehab are] group-centered with the veterans and they’re from all branches…you have a lot of shared life experiences…you build that camaraderie with the group…you form bonds.” (10026).

Along the same line, those who participated in Alcoholics Anonymous (AA) valued having veterans in the group and a fellow veteran as a sponsor to support them in their recovery. As two participants highlighted,

“I like being around other veterans that same way I was in doing the AA meetings at [location] VA. That’s what was really great about that, because we’re all veterans in there. I went to an AA meeting couple days ago… we had a, actually majority of us in there were veterans. But I just hate being intertwined with civilians when they trying to talk about their stuff, not get it. Everybody has problems. Civilians have problems too. But it just—it feels like there’s this awkwardness in the room where there’s some of us that get each other and the others don’t.” (10019).

“I go to AA, usually about five times a week. … I have a sponsor and he’s a fellow veteran. He was a [service branch name]. I was in the [different service branch name], so we kid each other a lot about that… [H]e’s been real helpful getting me through this and just being a friend.” (10024).

Not least of all, veteran service organizations and officers were also named as important facilitators, helping some participants gain access to the VA healthcare system. As one participant discussed,

“I went into the VSO office really intoxicated and I told them, ‘Look, one of my friends, you know, encouraged me to go and find you.’ There’s a VSO that was worth the shit, that saw what was going on and he grabbed me. He said, ‘I’ll try to get you in the system,’ and then that weekend I went and got a DUI…and that really expedited my paperwork. … [Doctor name] and [VSO officer name] were the two people that worked together to really make sure my shit was squared away.” (10028-1).

Although several participants reported being estranged from certain family members, spouses and family were nonetheless cited by several others as playing an important role in helping them to significantly reduce or limit their alcohol intake, and to access treatment:

“[My wife] keeps [the alcohol in the house] locked up…she’ll mix me a drink once in a while…and they’re nothing strong or anything. … [The drink] gets my mind off [the chronic back pain] for a while, and I can actually tolerate it for a few hours. … So, she limits me…it works pretty good.” (10025).

“[M]y wife was definitely vital in my receiving care, as far as she’s the one who made the phone calls for me. She really…saved my life in a lot of ways.” (10006).

“[My parents] worried about me in that apartment because…I was making good money at this job, but it was causing me more stress because of dealing with these idiots. And then, that was driving the drinking up even more. [The said] it would be a really good idea [to] move down with them because they wanted me to have a better support system” (10019).

## Discussion

Alcohol use disorder (AUD) is highly prevalent and carries a high mortality risk in veteran populations. Despite this, and the elevated frequency of alcohol withdrawal syndrome (AWS) admissions to VA hospitals, there is limited published data on veterans’ views or experiences seeking treatment for AWS, often referred to as “detox.” We aimed to understand veterans’ experiences during hospitalizations for AWS and the care they received for alcohol use disorder after discharge. The study was carried out among patients at two highly rural-serving VA hospitals that were selected as part of a quality improvement effort.

Participants provided useful insights into their views on alcohol use and motivations for drinking, many of which were associated with comorbid conditions, including chronic pain and PTSD, often linked to military service and combat experiences. The interview data aligned with prior studies identifying coping-related motives for increased alcohol use in patients with PTSD, like the perception that alcohol could decrease trauma-related thoughts and emotions (see, e.g., Zaso et al., 2024). According to our findings, participants who relied on alcohol to manage chronic pain symptoms often felt like pain medications lack efficacy. Similarly, those with co-existing pain and mental and alcohol use disorders often did not believe that a treatment to address co-occurring needs existed. These findings, which are worth considering by clinicians and researchers, agree with previous qualitative research indicating general dissatisfaction among patients with AUD seeking relief from symptoms associated with comorbid conditions (McCallum et al., 2015).

Findings from this study also identified several key factors influencing hospital admission and need for additional treatment services. These factors included acute withdrawal symptoms (e.g., seizures, hallucinations, delirium tremens), worsening of cognitive and motor functioning and other alcohol-related health complications (e.g., alcohol-induced hepatitis), adverse occupational and legal consequences (e.g., job loss or a DUI arrest), and social isolation and psychological distress caused by strained family dynamics. Earlier studies in patients seeking AUD treatment found a link between willingness to engage in treatment, the severity of mental health symptoms, and drinking-related consequences (Blume et al., 2006; Morris et al., 2018). Importantly, they found the severity of mental health problems was also associated with a higher risk of ongoing AUD. Our findings underscore the importance of multidisciplinary treatments for AUD in veteran populations addressing underlying mental health conditions and other causes of alcohol misuse as a prerequisite for successful AUD treatment.

Veteran perception of premature discharge was a common theme, often related to ongoing untreated symptoms or veterans’ own perceived time needed for recovery. This could stem from inadequately treated AWS or a mismatch between the patient’s goals and those of the treating physicians. Provider and hospital objectives are to address the immediate life-threatening issues and discharge the patient as safely and quickly as possible. Mild AWS does not require inpatient treatment and may last for up to a week (ASAM, 2020). AUD-associated derangements in sleep and mood may last for weeks to months (Drummond et al., 1998; Gopaldas et al., 2025). VA hospitals may also have a lower threshold for admission and monitoring of alcohol intoxication and withdrawal, given their emphasis on treating AUD and suicide prevention in this population. Ultimately, this might result in more opportunities for AUD treatment for veterans. The VA might consider reviewing options to align treatment with patient goals, given the negative outcomes associated with perceived premature discharge in this study.

One critical contribution of our study is the insight into veteran participants’ experiences of stigma. Studies show that AUD is considered to be among the most stigmatized mental disorders (Kilian et al., 2021), and individuals with AUD are less likely to seek alcohol treatment services if they perceive discrimination (Keyes et al., 2010). Although it is very likely veterans in our sample experienced AUD-related stigma outside of treatment settings, we focused only on their reports of external stigma and discriminatory treatment within the healthcare setting (Rehman et al., 2024).We found stigma about AUD was commonly experienced by veterans in both VA and non-VA hospital settings, particularly when undergoing treatment for AWS. Participants reported experiencing diminished empathy and discriminatory treatment, and being made to feel more responsible for their condition than other patients with chronic medical conditions (Kilian et al., 2021). Participants also reported cases where clinicians used unfavorable language toward or around them (e.g., using demeaning labels such as alcoholic or a drunk), which contributed to low self-esteem and a reduced willingness to seek treatment (Schomerus et al., 2011). Notably, some veterans highlighted that AUD-related stigma was more pronounced in non-VA (or community) hospital settings. The findings demonstrate that stigma is highly counterproductive and represents a major barrier to accessing treatment (Carrieri et al., 2022; Mekonen et al., 2021; van Boekel et al., 2013; Wallhed Finn et al., 2014), particularly in rural areas with limited hospital options. Furthermore, stigma may increase the burden of AUD and other alcohol-related diseases (Schomerus et al., 2022).

The findings underline the need for more support and education to reduce clinician stigma, prevent compassion fatigue among healthcare professionals, and provide better care to veterans with AUD (Wakeman et al., 2016). One strategy that has been successful in changing provider attitudes towards patients with SUD is the use of the patient voice in educational interventions (Schlaudecker et al., 2020). To decrease clinician stigma, the VA could implement educational information sessions and visual messaging campaigns featuring veterans living with AUD (Kennedy-Hendricks et al., 2022). We also recommend the VA designate more resources to address knowledge deficits regarding AWS and AUD treatment. Future studies that evaluate AUD-related stigma in VA and clinicians’ aptitude and willingness to work with veterans with AUD are also warranted. Addressing AUD-related stigma in VA is likely to have a positive impact on veterans with AUD living, particularly in rural areas, where misconceptions about treatment, a shortage of specialized services, and a relative lack of privacy/anonymity in healthcare settings due to the small size of the population act as additional barriers to treatment (Browne et al., 2016; Fortney et al., 2004; Richard et al., 2020).

Geographic distance and the travel burden of attending residential treatment many hours away from home was another major and unique issue reported by the participants in our study, in addition to the need to spend prolonged periods separated from their family or home. This was a challenge affecting both participant sites, and that differs from previous literature. Findings revealed that participants frequently traveled long distances across state lines to access residential rehabilitation treatment programs (RRTP), in part due to the large VA catchment areas encompassing the hospitals, clinics, and treatment programs that served them. Participants also consistently mentioned long wait times for accessing RRTPs after admission for AWS. A shortage of RRTPs and limited capacity in veteran’s catchment areas contributed to long wait times, but so did the excess demand for RRTPs managed and staffed by VA, which were perceived as providing better care to veterans. These findings reflect the additional burdens that veterans living in rural areas endure to access treatment. Furthermore, veterans would prefer to go directly from inpatient hospital treatment to residential rehabilitation when needed, feeling that returning home or to outpatient care poses a greater risk of returning to drinking. Direct discharge to a VA RRTP would be ideal, but these programs are limited and highly sought after. In addition, the limited numbers of facilities across the country that offer these services increases the travel burden and barriers to care. Though the Maintaining Internal Systems and Strengthening Integrated Outside Networks (MISSION) Act of 2018 (Public Law 115–182, 132 Stat. 1393) authorizes the VA to purchase care in the community, if the VA cannot provide RRTP services within 72 h for priority patients or 30 days for routine admissions, the availability and quality of rehabilitation care in rural areas is unknown (Reinkemeyer, 2025). This is an area in which future studies could evaluate the impact of different care modalities, such as telehealth or daily walk-in clinics, to bridge this gap in care.

Treatment cost is often cited as an individual barrier in the qualitative literature on treatment seeking for AUD (Venegas et al., 2021), however, we did not find this to be the case in our sample. When participants cited financial concerns as a barrier to treatment, they mainly referred to the cost of transportation, i.e., gas or the cost of a taxi ride to the hospital when no one was available to drive them or when driving themselves to the hospital would put them and/or others at risk. This difference between our study and previous literature is context dependent, given that VA benefits will often cover access for SUD treatment at limited or no cost for the veterans, as opposed to community settings where insurance coverage for such treatments is highly variable among payers.

Although clinician stigma about AUD was commonly reported, participant perspectives on treatment nevertheless identified clinician support as an important facilitator to engaging in treatment. Clinicians were identified by participants as playing a crucial role in helping patients access treatment and build a positive sense of self, which has been shown to be a key aspect of recovery (Hill & Leeming, 2014). Veterans credited VA clinicians who were genuinely supportive, consistently concerned, and communicated in a non-judgmental manner. The findings highlight the positive impact of building trust and engaging patients in developing a positive self-esteem, and illustrate why clinicians should view these actions as a critical priority (Wangensteen & Hystad, 2022).

Veteran perspectives on treatment also noted that a supportive social network was crucial to their engagement in AUD treatment. Reflecting the literature on the role of the family in AUD recovery, we found spouses and other family members facilitated access to treatment and helped veterans reduce drinking and manage cravings; and that meaningful connections to family were key motivators to engaging in treatment (McCrady & Flanagan, 2021). Participants also identified peer support from veterans as playing an important role in coordinating or re-establishing care with the VA, a finding that mirrors the literature on peer recovery support (Eddie et al., 2025), which found that peer support improves engagement and treatment retention. A recent study of veterans with co-occurring anxiety disorders or PTSD and unhealthy alcohol use found building community among others in recovery was a facilitator for the treatment of both disorders (Ecker et al., 2025). Given these findings, the VA should examine ways to further foster community building among veterans and leverage peer-support programs to address barriers to care.

The limitations of this study include the small purposive sample of veterans admitted to a rural-serving VA hospital with an AWS-associated ICD-10 code, and an all-White and predominantly male sample, for which our results might not be generalizable to other patient populations or healthcare systems. These samples might over-represent veterans with a particularly positive or negative experience, (self-selection bias) and those with more serious AUD or AWS, as those were the patients admitted to the hospital. Given that interviews were conducted weeks or months or even a year after hospital admissions, participants might have recall bias or provide an incomplete recount of their experiences.

## Conclusion

This study presented several themes that emerged from interviews with rural veterans who had an admission for alcohol withdrawal syndrome, and their experience with alcohol use disorder (AUD) care after discharge. Rural veterans reported similar reasons for drinking as previously described in the literature including co-occurring conditions, most commonly pain, PTSD, and negative affect. Veterans experienced structural barriers and provider stigma and often felt pressured to discharge before they felt ready. This occurred at all facilities, but less so at VA hospitals. Veterans felt like the transition from inpatient to outpatient treatment was handled better at VA hospitals, and the VA appeared more VA appeared more invested in their overall care. Veterans would prefer immediate access to residential treatment for AUD, yet long wait times and travel distances often limit access to this care, Taken together, our findings provide additional opportunities to improve AUD care in veterans. Ideally, multidisciplinary teams with a coordinated, standardized, and evidence-based approach to AUD care, including reducing stigma, providing a positive treatment environment, timely initiation of medications for AUD treatment, and expediting post-discharge access to rehabilitation treatment programs will become the norm within VA care, similar to how Centers of Excellence for other conditions like cancer care or stroke have optimized treatment and outcomes for illnesses with high morbidity and mortality.
